# Embodied Cross-Domain Intelligence in Biomedical Microrobots: A Review

**DOI:** 10.34133/cbsystems.0546

**Published:** 2026-05-14

**Authors:** Zongcai Tan, Lan Wei, Kangyi Lu, Dandan Zhang

**Affiliations:** Department of Bioengineering, Imperial-X AI Initiative, Imperial College London W12 0BZ, London, UK.

## Abstract

Microrobots are emerging as transformative tools for biomedical applications, including minimally invasive diagnostics, targeted therapy, and microsurgical intervention. However, achieving reliable performance and adaptation to diverse tasks and environments requires capabilities that exceed any single form of intelligence. Here, we introduce embodied cross-domain intelligence, a framework for synergistic coupling across physical, biological, computational, and human intelligence, enabling multifunctional, collaborative, and adaptive microrobotic behavior in dynamic, safety-critical biological settings. Unlike prior reviews that address these intelligence domains in isolation, this review aims to provide a unified framework, outlining each domain’s principles, recent advances, and limitations; analyzing the interfaces that foster synergy; and mapping representative domain combinations to major biomedical applications. We further identify core challenges in integration, control, safety, and validation and outline future research directions to accelerate clinical translation. By framing biomedical microrobot development as a cross-domain synergy challenge, this review aims to guide interdisciplinary efforts toward systems capable of executing complex, multistage tasks across their operational life cycle. The associated project is available on the online project page (https://nuounuou.github.io/Embodied-Cross-Domain-Intelligence-in-Biomedical-Microrobots-A-Review/).

## Introduction

Microrobots are miniaturized devices, typically untethered, that perform tasks such as targeted payload delivery, tissue engineering, and microsurgical intervention [1,2]. Powered by external fields or local energy, they move through bodily pathways to inaccessible regions, enabling targeted therapy, diagnostics, and minimally invasive surgery in vivo [3]. In vitro studies have shown capabilities including single-cell manipulation, tissue sampling, and controllable drug release in microfluidic chips [4]. Building on this basis, biomedical microrobotics, combining robotics, materials science, biology, and medicine, has advanced rapidly [5].

Despite these advances, most experimentally demonstrated microrobots are still designed for single, well-defined tasks [5,6]. They are actuated by external fields and follow pre-programmed or operator-defined motion, with only limited feedback or autonomy [7–9]. As a result, their ability to sense, learn, and adapt to the highly dynamic and heterogeneous environments of living systems remains restricted. Achieving reliable performance in complex biological contexts therefore requires the synergy of intelligent capabilities that allow perception, decision-making, and adaptation [10].

Early efforts have introduced distinct single-domain intelligence into microrobotic systems. Physical intelligence (PI) uses material properties, structural design, and energy transduction for adaptability and self-organization [11]. Biological intelligence (BI) harnesses living cells and organisms for actuation, sensing, and self-healing [12]. Computational intelligence (CI) provides algorithms for control, perception, and learning to enable autonomy [13]. Human intelligence (HI) supplies expertise and intuition to guide and supervise operation [14].

However, each intelligence domain alone is insufficient for robust performance in real biomedical environments. Existing reviews offer insights into individual aspects such as materials, actuation, control strategies, and biomedical applications [3,11,15,16] but rarely examine how different forms of intelligence interact or how their combination enhances microrobotic capability. We therefore propose embodied cross-domain intelligence, a unified framework that integrates PI, BI, CI, and HI to endow microrobots with multifunctional, adaptive, and collaborative behavior for safe and effective operation in dynamic, safety-critical biological environments.

**Fig. 1.**
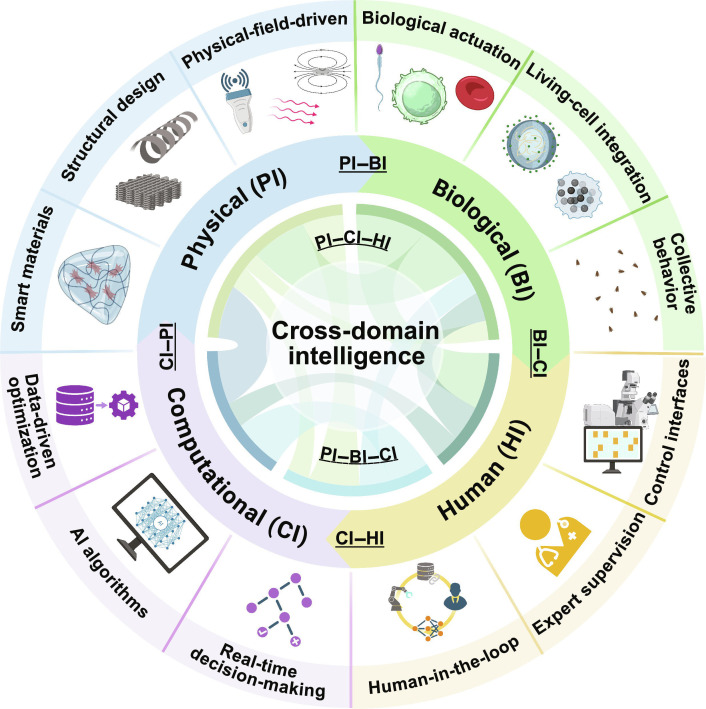
Overview of embodied cross-domain intelligence in biomedical microrobots. Physical (PI), biological (BI), computational (CI), and human intelligence (HI) are co-designed and coupled through domain interfaces (e.g., PI–BI, BI–CI, CI–HI, and CI–PI), enabling capability transfer and higher-order synergies for adaptive microrobotic operation in complex biomedical environments. The outer ring highlights the representative elements of each domain, including smart materials and structural/field-driven actuation (PI); biological actuation and living-cell integration (BI); data-driven optimization, artificial intelligence (AI) algorithms, and real-time decision-making (CI), and human-in-the-loop supervision and control interfaces (HI).

This review aims to bridge this gap by presenting, for the first time, a unified examination of embodied cross-domain intelligence in biomedical microrobots and its practical implications. Here, we mainly consider typically untethered microrobots, while “microrobotic systems” can also cover guided microtools and partially tethered platforms. We define embodied cross-domain intelligence as the synergistic capabilities that emerge from the interplay among PI, BI, CI, and HI in microrobotic systems. Notably, “cross-domain” in this review emphasizes mechanism-level co-design and capability transfer across distinct intelligence domains (e.g., translating biological strategies into embodied structures and/or computational policies) and should be distinguished from CI-centric multimodal intelligence that primarily fuses heterogeneous modalities at the input/data level. Fig. 1 summarizes the proposed embodied cross-domain intelligence framework and representative interfaces among PI, BI, CI, and HI. We first outline the principles and representative advances of each intelligence domain and then analyze the interfaces that enable their cooperation. We further map domain combinations to major biomedical applications. We then discuss 4 core challenges in integration, control, safety, and validation and propose a staged research road map to accelerate clinical translation. By framing microrobotic development as a cross-domain synergy challenge, this review aims to guide interdisciplinary efforts toward the next generation of intelligent, multifunctional, and clinically deployable microrobotic systems.

## Intelligence Domains in Microrobotic Systems

Microrobots can express intelligence through mechanisms embodied in their physical structure, biological components, computational frameworks, and human interaction. These mechanisms are categorized into 4 domains: PI, BI, CI, and HI. Each domain contributes distinct yet complementary capabilities, and their integration defines the foundation of embodied cross-domain intelligence in biomedical microrobotics.

### Physical intelligence

#### Definition and rationale

PI (as summarized in Table 1, rows 1 and 2) refers to the capability of physically encoding sensing, actuation, control, memory, logic, and adaptation directly into the body of an agent, independent of a central digital brain or external controller [17]. At micro- and nanoscales, where traditional actuators and power sources are impractical, materials and morphology become the primary sources of intelligent behavior [18]. A stimulus-responsive behavior is considered PI only when it implements a task-structured mapping from environmental cues to functional actions that reduces external control burden; purely passive compliance or generic deformation without such selective structure is treated as material functionality rather than PI. This domain forms the basis of autonomous microrobotics in biomedical contexts by directly coupling environmental stimuli with mechanical response [19,20].

**Table 1.** Summary of intelligence domains in microrobotic systems

Domain	Role	Mechanisms	Key challenges	Representative tasks	Refs.
PI	Material-driven autonomy	Stimulus-responsive materials (e.g., hydrogels and liquid crystals), magnetoelastic composites	Limited selectivity and sensitivity to physiological noise (e.g., off-target activation)	Targeted drug release (pH/temp triggered), tissue biopsy	[21–23,25,26]
	Structure-driven autonomy	Bioinspired geometries (e.g., helical tails), shape-morphing structures, surface textures	Difficulty integrating multiple functions within constrained geometries	Vascular swimming in viscous blood	[27]
BI	Integration of living cells/organisms for actuation, sensing, and adaptability	Biohybrid robots using iPSCs, neutrophils, *Escherichia coli*, or platelets for targeted delivery and chemotaxis-based navigation	Biological variability, short lifespan, limited controllability in vivo	Tumor-homing delivery, chemotactic cargo transport	[33–36]
CI	Algorithm-driven learning, perception, and control enabling autonomy and adaptation	CNNs for perception, RL and IL for navigation and task learning, digital twins for simulation and control	Onboard computing limits, data scarcity, need for safety assurance in medical contexts	Image-based depth/pose estimation, closed-loop navigation	[13,41,44,47,114]
HI	Cognitive guidance and expert control through human–robot interfaces	Teleoperation, haptic feedback, AR/VR interfaces, shared-control frameworks	Latency, interface design limitations, limited situational awareness in 3D tasks	VR-guided vascular steering, remote micromanipulation	[14,44,46,49]

PI, physical intelligence; BI, biological intelligence; CI, computational intelligence; HI, human intelligence; iPSCs, induced pluripotent stem cells; CNNs, convolutional neural networks; RL, reinforcement learning; IL, imitation learning; AR, augmented reality; VR, virtual reality; 3D, 3-dimensional

#### Mechanisms and representative systems

Stimulus-responsive materials and bioinspired structures embody PI by reacting predictably to changes in the environment. Hydrogel-based microrobots can release drugs in response to the acidic conditions of tumor microenvironments [21–23], thermo-triggered untethered microgrippers can self-close at physiological temperature to excise tissue for biopsy [24], and magnetoelastic soft composites enable wireless locomotion together with shape reconfiguration under magnetic actuation [25,26]. Bioinspired geometries such as helical, flagellum-like tails improve propulsion efficiency and stability in viscous, blood-like media [27]. These examples demonstrate how material composition can act simultaneously as a sensor, an actuator, and a controller.

#### Challenges and outlook

PI offers robust, low-energy operation but has limited selectivity and multifunctionality in complex physiological environments [28]. Integrating multiple functions in submillimeter devices therefore remains difficult. Advances in multiresponsive biodegradable materials, reconfigurable structures, and computationally optimized designs are expected to strengthen PI as the material basis for autonomous microrobots capable of responding intelligently to their biological milieu [20].

### Biological intelligence

#### Definition and rationale

BI (as summarized in Table 1, row 3) arises from integrating living cells/organisms or biologically derived components as functional elements, endowing intrinsic sensing, actuation, and adaptive behaviors [29,30]. This domain leverages the inherent efficiency, selectivity, and self-healing capabilities of biological systems to enable microrobots to operate effectively within the dynamic and heterogeneous environments of the human body [31,32].

#### Mechanisms and representative systems

Biohybrid microrobots combine living organisms with synthetic structures to achieve controlled locomotion and targeted delivery. Examples include induced pluripotent-stem-cell-based microrobots with tumor-homing abilities for precise nanoparticle delivery [33], neutrophil-driven carriers that exploit chemotaxis for targeted drug release [34], and *Escherichia coli*-powered hybrids that navigate hypoxic regions through natural taxis [35]. Platelet-based systems use receptor-mediated adhesion for site-specific tumor targeting [36]. These strategies demonstrate how biological entities can serve as intelligent subsystems capable of sensing and responding to complex biochemical gradients.

#### Challenges and outlook

Autonomy in BI-based systems depends on robust in vivo function of living components in complex physiological settings. Variability between batches, limited lifetime, and environment-dependent taxis hinder reproducibility, scale-up, and regulatory translation [29,37]. Immune interactions and heterogeneous biochemical cues can alter cell behavior over time, affecting targeting and safety [30]. Future work will use genetic engineering and synthetic biology to program taxis and payload release [38] and combine biohybrids with closed-loop sensing and control to stabilize navigation. Standardized encapsulation, storage, and benchmark tests are needed for reliable performance. This will also improve quality control and support more predictable clinical outcomes.

### Computational intelligence

#### Definition and rationale

CI (as summarized in Table 1, row 4) encompasses the use of data-driven and algorithmic approaches that enable microrobots to perceive, plan, and act adaptively in dynamic and uncertain environments [13,39]. This domain complements the passive adaptability of PI and BI by introducing active learning and decision-making capabilities that support autonomy and precision.

#### Mechanisms and representative systems

CI integrates perception, control, and learning modules, although current validation levels vary from training in simulation to in vitro demonstration. Vision-based systems using deep learning allow the estimation of microrobots’ depth, pose, and position [40]. While highly effective, these are primarily validated in vitro microscopy settings [41]. The sim-to-real gap refers to the degradation in perception and performance when models are transferred from simulation to real-world settings. To mitigate this gap, domain adaptation techniques, often combined with simulation-based data augmentation, are increasingly employed [13,42,43]. Reinforcement learning (RL) and imitation learning algorithms enable adaptive navigation under uncertainty [44,45]. Most current RL policies are trained in simulation due to data requirements, with recent efforts successfully demonstrating transfer to in vitro microfluidic environments [46]. Advanced platforms now utilize digital twins (virtual replicas synchronized with physical systems) to accelerate algorithm training and improve real-world transferability [47].

#### Challenges and outlook

CI-driven systems face constraints in computational resources, data availability, and real-time safety assurance. Onboard processing is often limited by power and computational budgets, and biomedical datasets remain scarce. A critical bottleneck is developing perception algorithms robust to the low signal-to-noise ratios and artifacts typical of realistic clinical imaging. Future progress will depend on physics-informed learning, federated data sharing, and interpretable artificial intelligence (AI) architectures that ensure safety and transparency.

### Human intelligence

#### Definition and rationale

HI (as summarized in Table 1, row 5) represents the cognitive and perceptual contributions of clinicians and expert operators in the control, real-time monitoring, and intervention of microrobots [14,48]. By combining human intuition with robotic precision, this domain facilitates human-in-the-loop (HITL) systems that enhance reliability, safety, and ethical oversight in biomedical applications. Here, HITL is used as an umbrella term; shared control is a typical HITL implementation.

#### Mechanisms and representative systems

HI manifests through teleoperation, haptic feedback, and augmented- or mixed-reality interfaces that integrate human expertise with robotic control. Haptic shared control has been shown to improve task precision and reduce completion time during 3-dimensional (3D) navigation of microrobot swarms [14]. Immersive visualization tools, such as patient-specific augmented-reality (AR) interfaces, enhance situational awareness during real-time manipulation of microrobots within vascular networks [49]. Adaptive autonomy frameworks combining deep RL with workload monitoring dynamically balance control between human and machine, reducing user fatigue and improving accuracy [44].

#### Challenges and outlook

Limitations in visualization, haptic sensing, and latency currently hinder fine control and limit reliability in clinically relevant settings [50]. Addressing these challenges requires advances in high-fidelity imaging, ergonomic interface design, and adaptive assistance algorithms that compensate for human tremor and cognitive load [44,47]. Ultimately, HI provides the supervisory layer that aligns machine operation with human judgment and clinical intent, ensuring safety and trust in intelligent microrobotic systems.

## Cross-Domain Synergies: Integrating Multiple Intelligences

Embodied cross-domain intelligence coordinates multiple intelligence domains to achieve functions unattainable by any domain alone. A cross-domain intelligent microrobot can combine responsive materials (PI) for field-driven propulsion, biohybrid actuators (BI) for environmental sensing and swarm collaboration, computational controllers (CI) for autonomous navigation from real-time task and environment perception, and human expertise (HI) for high-level supervision and safety. In this section, we first discuss the most prevalent cross-domain couplings and then survey representative systems that integrate multiple domains within a single biomedical workflow.

### PI–BI synergy

**Fig. 2.**
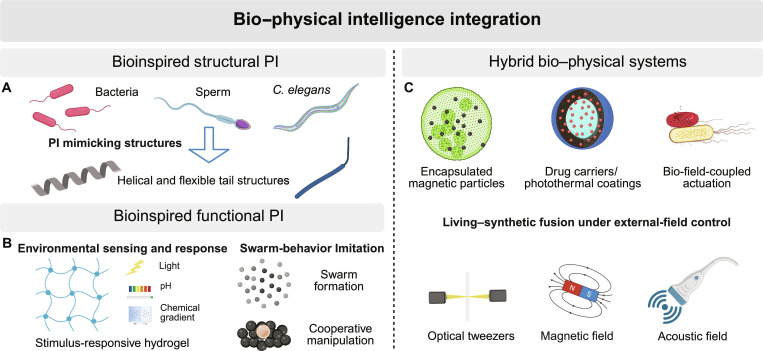
Conceptual summary of biological intelligence (BI)–physical intelligence (PI) synergy mechanisms: (A) bioinspired structural PI: geometry-encoded locomotion and adaptability via helical and flexible tails; (B) bioinspired functional PI: stimulus-responsive hydrogels and swarms enabling environment-dependent actuation and cargo handling; and (C) hybrid bio–physical systems: biohybrid microrobots combining living cells or microorganisms with synthetic carriers for controllable, targeted therapy.

The integration of PI and BI combines complementary strengths that are limited when each domain operates in isolation. PI contributes engineered responsiveness and actuation yet often lacks intrinsic compatibility with complex biological environments. BI, by contrast, offers natural adaptability, self-propulsion, and environmental sensing but is constrained by limited controllability and stability. Integrating these domains enables microrobots to couple sensing, actuation, and adaptation within a single embodied system, bridging the gap between engineered design and biological function (Fig. 2).

#### Bioinspired structural PI

##### Definition and rationale

The first mechanism of PI–BI synergy is bioinspired structural design, in which PI is realized through the imitation of biological geometries and morphologies (Fig. 2A). This approach translates nature’s efficient mechanical strategies into manufacturable microscale architectures that enhance locomotion and adaptability in complex environments. For instance, helical and flexible tail structures inspired by bacterial and sperm flagella enable propulsion at low Reynolds numbers. Helical magnetic swimmers achieve directional motion under rotating magnetic fields without explicit control logic [51]. Magnetically driven nickel-coated graphene-based helical microrobots have achieved angular drift below 0.5° and swimming speeds up to 2.6 body lengths per second, illustrating geometry-encoded precision [52]. Similarly, Huang et al. [53] developed a self-folded hydrogel swimmer with a flagellum-like tail, where body–tail coupling and wobble-induced precession enhance propulsion efficiency. At the surface level, gecko-inspired anisotropic friction textures enable microrobots to crawl in both liquid and dry conditions through direction-dependent frictional gait transitions [54]. These examples highlight how morphology itself can serve as an intelligent medium for motion and adaptation in microrobotic systems.

##### Biomedical applications

In terms of application classes, bioinspired structural PI most often contributes to minimally invasive surgery and endovascular intervention and targeted drug and biologic delivery, because geometry-encoded propulsion and contact mechanics can reduce control burden while improving access in confined lumens and heterogeneous tissue interfaces [55,56]. Recent endovascular demonstrations show that helical magnetic robots can be engineered to exploit their morphology for intravascular progression and steering, thereby improving reachability in tortuous vascular pathways that challenge conventional catheterization workflows [55]. Related helical robot designs have also been verified in vessel-mimicking settings as magnetically guided micro-guidewire platforms, supporting controlled advancement and directional maneuvering in constrained channels where low-Reynolds-number locomotion and wall interaction dominate the task physics [56]. Across these tasks, the performance benefit is not merely that the robot is “magnetically actuated”, but that the bioinspired helical architecture itself embodies part of the control and interaction strategy, which directly aligns with the structural PI mechanism described above [55,56].

#### Bioinspired functional PI

##### Definition and rationale

The second mechanism involves bioinspired functional PI, where microrobots emulate the environmental sensitivity and adaptive behaviors of living organisms (Fig. 2B). Stimulus-responsive hydrogels deform under biochemical triggers and enable cargo release and motion [57]. Chemoresponsive hydrogels can swell, shrink, or stiffen upon biochemical triggers, allowing controlled cargo release or motion. Embedded enzymes convert gradients of pH, glucose, or urea into logic-gated motion and targeted delivery [58–60]. Xin et al. [61] developed a femtosecond laser fabrication method to integrate hydrogels with metal nanoparticles, producing multijoint microactuators that mimic humanlike motion and perform precise microcargo manipulation. Functional bioinspiration thus embeds adaptability directly into materials, allowing microrobots to respond intelligently to complex surroundings. At the collective level, bioinspired swarm microrobots can transition between liquid-like, vortex, or rod-shaped configurations, enabling adaptive navigation and cooperative manipulation in changing environments [62,63].

##### Biomedical applications

From an application-mapping standpoint, bioinspired functional PI most directly supports targeted drug and biologic delivery and immunomodulation and cell therapy support, because it turns endogenous biochemical cues into embodied locomotion, retention, or release behaviors that are difficult to reproduce with external actuation alone [60,64]. A representative in vivo therapeutic example is a urease-powered nanomotor loaded with a stimulator of interferon genes agonist for bladder cancer immunotherapy, where enzyme-enabled propulsion and swarming in urea-rich bladder conditions improves tissue penetration and retention after intravesical instillation and yields a reported antitumor inhibition of 94.2% together with an 11.2-fold increase of CD8+ T-cell recruitment compared with phosphate-buffered saline [60]. In parallel, magnetically driven hydrogel microrobots have been validated for drug transport and on-site release in tumor-related settings, using chemically responsive dissolution as a functional trigger that couples local biochemical environment to therapeutic deployment rather than relying on passive diffusion alone [64]. These studies instantiate the same functional principle emphasized in this subsection, namely, that bioinspired stimulus-to-action transduction embeds task-relevant adaptation into the material and chemical interface with the biological milieu [60,64].

#### Hybrid bio–physical systems

##### Definition and rationale

The third mechanism involves hybrid bio–physical systems, which integrate living organisms or biological templates with synthetic materials to achieve advanced functionalities (Fig. 2C). Biological components such as red blood cells, stem cells, sperm, bacteria, and algae inherently provide motility, biocompatibility, and environmental sensing [29]. Incorporating magnetic coatings, photothermal layers, or drug-loaded shells can add external controllability and multifunctionality. Wu et al. [65] developed erythrocyte-based microrobots that codeliver drugs and immune molecules while modulating programmed cell death ligand 1 expression, enhancing T-cell-mediated cancer therapy. Li et al. [66] constructed an alga-based microrobot coated with macrophage membranes to capture and neutralize proinflammatory cytokines, offering a noninvasive treatment for inflammatory bowel disease. Chen et al. [67] introduced the AlgaeSperm microrobot, combining magnetic algae with a sperm-like head–tail structure for high-speed propulsion and chemo–photothermal synergistic therapy. These biohybrid systems unite the adaptability of living organisms with the precision of engineered design, marking a key step toward autonomous, programmable, and clinically relevant microrobotic platforms.

##### Biomedical applications

In terms of application classes, hybrid bio–physical systems most consistently map to targeted drug and biologic delivery and immunomodulation and cell therapy support, because living carriers contribute biocompatible motility and in situ interactions, while engineered coatings and payload interfaces provide task-specific capture, therapy, and controllability [66–68]. For inflammatory bowel disease, an alga-based biohybrid robotic system has been shown to actively neutralize colonic cytokines by combining motile microalgae with macrophage-membrane-coated nanoparticles, thereby coupling biological dispersion with engineered cytokine-binding interfaces in a disease-relevant gastrointestinal setting [66]. For lung metastasis treatment, motile algae have been used to locally and actively deliver drug-loaded nanoparticles in the lung, where the biohybrid embodiment supports deep-lung distribution while synthetic nanoparticle interfaces implement chemotherapy payload carriage and release, providing a concrete example of PI and BI co-design for localization and retention in vivo [68]. For tumor-targeted chemo–photothermal therapy, microalga-based soft magnetic microrobots with sperm-like architectures have been reported to enable targeted anticancer action after drug loading, illustrating how biotemplated bodies and engineered magnetic structures jointly support propulsion and therapy execution rather than merely coexisting in the same platform [67].

Collectively, PI–BI synergy exemplifies how material embodiment and biological adaptability can converge to create intelligent microrobots capable of operating effectively in physiological environments. Structural and functional bioinspiration provide the foundation for movement and responsiveness, while hybrid living–synthetic systems extend these capabilities toward therapeutic and diagnostic applications. Continued advances in biocompatible smart materials, interface engineering, and hybrid fabrication are expected to enable truly autonomous and sustainable microrobotic systems within the human body.

### PI–CI synergy

**Fig. 3.**
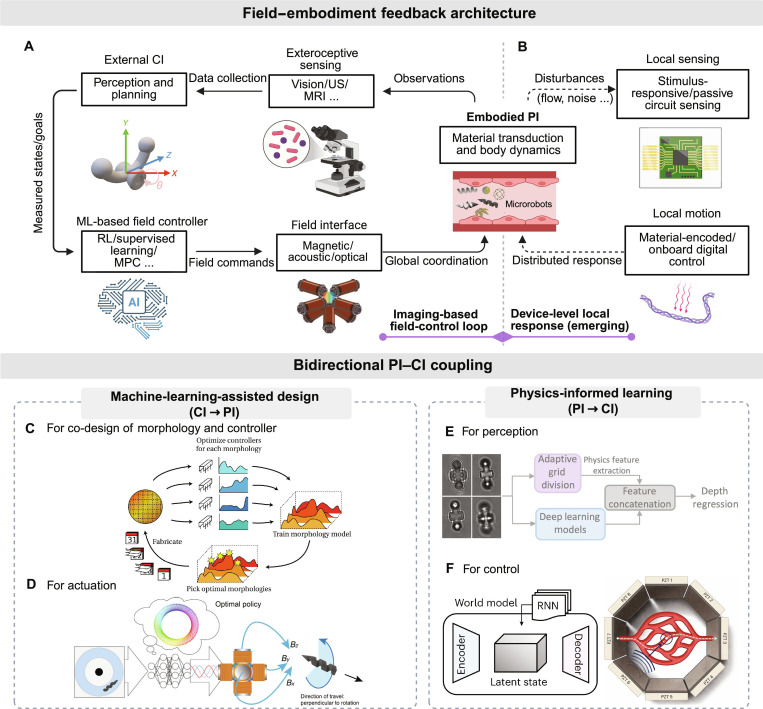
Physical intelligence (PI) and computational intelligence (CI) synergy mechanisms in biomedical microrobotics. (A) Imaging-based field-control feedback: external imaging/tracking (e.g., vision/ultrasound [US]/MRI) estimates the microrobot state around the device, enabling the CI controller to update magnetic/acoustic/optical field commands in a closed loop. (B) Device-level local response (emerging): material-encoded stimulus–response behaviors, self-sensing readouts, and minimal onboard logic support local response and event-triggered behaviors that can augment external-field control. (C and D) CI-assisted embodiment (CI → PI): (C) Bayesian optimization co-designs morphology and controller to reduce fabricate–test cycles; (D) deep reinforcement learning (RL) learns magnetic-field control policies that drive autonomous microrobot swimming. (E and F) Physics-informed learning (PI → CI): (E) physics-based focus metrics regularize convolutional neural network (CNN) depth estimation of transparent optical microrobots; (F) learned world models and recurrent policies enable sample-efficient navigation of US microswimmers. Figure reproduced or reprinted from published sources as follows: (C) Reprinted with permission from Ref. [74]. Copyright 2019 IEEE; (D) Reproduced from Ref. [51] under Creative Commons license CC BY 4.0; (E) Reprinted in part with permission from Ref. [75]. Copyright 2025 IEEE; and (F) Reproduced from Ref. [46] under Creative Commons license CC BY 4.0.

The synergy of PI and CI allows microrobots to couple material embodiment with data-driven reasoning. This synergy improves actuation precision, adaptability, and robustness in complex biomedical environments where analytic models are limited. As summarized in Fig. 3, PI–CI synergy mainly appears as (a) a hierarchical field–embodiment feedback architecture for closed-loop actuation and (b) bidirectional learning links where CI optimizes embodiment (CI → PI) and physics priors regularize perception/control (PI → CI).

#### Field–embodiment feedback architectures

##### Definition and rationale

Field–embodiment feedback couples an algorithmic controller (CI) that updates external actuation fields with an embodied microrobot (PI) that transduces those fields into propulsion, deformation, or contact-mediated function (Fig. 3A). In most reported platforms, the actuation loop is closed through external sensing and tracking (e.g., microscopy, ultrasound, or MRI), which produces robot-centric state estimates in the vicinity of the device; these estimates form the observation for the CI controller, which then updates field commands at the next control step. This “sense–estimate–actuate” cycle is exemplified by learning-based field control: RL policies can directly map tracked states or task errors to coil-current commands for 3D magnetic positioning, achieving mean distance errors below 30 μm after a few control steps in representative demonstrations [69]. Likewise, for ultrasound microswimmers, Schrage et al. [70] used a vision-based tracker (Channel and Spatial Reliability Tracking [CSRT], ~33 fps) to extract the swimmer position $p_{s}\left[n\right]$ from each video frame, feed $p_{s}\left[n\right]$ into an RL policy $\pi \left(\cdot \right)$, and output the acoustic driving parameters (e.g., $V_{\mathrm{pp}}$ and $f_{0}$) that are applied to the piezoelectric transducer array before the next tracking update. In these systems, “local perception” is thus realized as local state estimation around the robot by external sensing, and the closed loop is completed when the estimated state is used to update the next field command.

A second, complementary line of work moves parts of sensing and decision closer to the microrobot body, enabling local response without requiring continuous global replanning (Fig. 3B). One route is to encode task-relevant stimulus–response mappings into materials and surface interactions, such that local environmental cues modulate function directly (e.g., rolling microbots whose traction can change via pH-mediated adhesion) [71]. Another route integrates microsystems and minimal digital logic to generate predesigned actuation sequences and simple command-following behaviors at the device level [72]. Relatedly, “self-sensing” concepts can encode the microrobot state into externally readable signatures for noninvasive monitoring, supporting supervisory updates or event-triggered interventions when combined with CI [73]. At present, these device-level capabilities most often augment externally sensed navigation rather than replacing it, but they provide a clear pathway toward faster local stabilization and richer field–embodiment feedback in future platforms.

#### Bidirectional PI–CI coupling

##### Definition and rationale

The second mechanism links PI and CI through learning over design and data (Fig. 3B to E). In the CI → PI direction, machine learning assists embodiment. Liao et al. [74] use hierarchical Bayesian optimization to co-design microrobot morphology and controller: batches of morphologies are fabricated in parallel, each device learns its own controller, and a surrogate model proposes new designs, reducing costly fabrication–evaluation cycles. Abbasi et al. [69] trained an RL policy that maps microrobot and target states directly to coil currents, enabling autonomous 3D positioning in nonlinear magnetic fields without explicit system identification. Complementarily, Behrens and Ruder [51] used soft actor–critic deep RL to learn coil-current magnitudes and phase offsets (from either state feedback or raw images), shaping time-varying magnetic fields that drive robust helical microrobot swimming in uncharacterized channels. In the PI → CI direction, physical knowledge informs perception and control. Wei et al. [75] fused physics-based focus metrics, computed on adaptive grids, with convolutional neural networks’ features to regress the depth of transparent optical microrobots from monocular images, achieving higher accuracy and strong data efficiency. Medany et al. [46] developed a model-based RL framework in which a learned world model and recurrent policy capture the latent dynamics of ultrasound-driven microswimmers, enabling sample-efficient navigation and collision avoidance in vascular-like channels.

##### Biomedical applications

In terms of biomedical application classes, bidirectional PI–CI coupling most naturally serves minimally invasive surgery and endovascular intervention as well as in vivo diagnostics and biosensing, because these scenarios demand that embodiment, sensing, and control be treated as a coupled design problem rather than independent modules. A representative example is an ultrasound-driven microrobot system that integrates learned latent dynamics with model-based RL to realize autonomous navigation and obstacle-aware motion primitives from image observations, directly supporting closed-loop operation in vessel-like pathways that are central to endovascular-style navigation tasks [46]. Complementarily, imaging-guided bioresorbable acoustic hydrogel microrobots exemplify how material embodiment and computational imaging feedback can be co-developed so that stable locomotion, anatomical tracking, and control authority remain mutually consistent, enabling repeatable navigation under clinically relevant ultrasound guidance [76]. At the human scale, magnetic microrobot navigation in hepatic artery anatomies further demonstrates that computational planning under embodied constraints can materially expand the feasible operating envelope for endovascular targeting strategies, aligning navigation physics, patient-scale disturbances, and procedural objectives within a single optimization loop [77].

Collectively, PI–CI synergy shows how coupling embodiment with computation can transform microrobotic performance: field–embodiment architectures enable closed-loop control across scales, while machine-learning-assisted PI and PI-informed CI reduce control complexity and data demands by sharing intelligence between materials and algorithms.

### BI–CI synergy

**Fig. 4.**
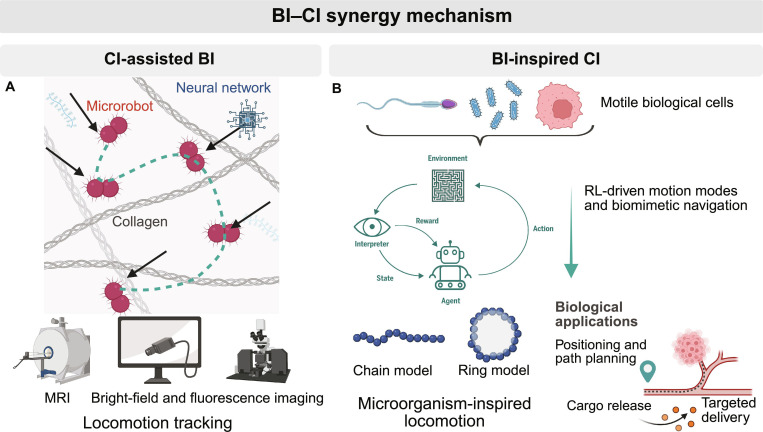
Synergy of biological intelligence (BI) and computational intelligence (CI) in microrobots. (A) CI-assisted BI: learning-based perception and imaging track biohybrid microrobots in dense matrices for locomotion control. (B) BI-inspired CI: reinforcement-learning algorithms trained on motile cells rediscover chemotactic navigation and microorganism-inspired locomotion, enabling positioning and path planning, cargo release, and targeted delivery.

The synergy between BI and CI in microrobotics is best understood as functional coupling across perception and control (Fig. 4). On the BI side, living components provide self-propulsion, environmental sensing, and biocompatibility; whereas on the CI side, learning-based perception and policy synthesis translate noisy observations into actionable commands in viscosity-dominated microflows (i.e., low-Reynolds-number regimes).

#### CI-assisted BI

##### Definition and rationale

Learning-based perception closes a long-recognized limitation in dense, low-contrast media where biohybrid agents are nearly indistinguishable from background structures (Fig. 4A). MEMTrack introduces a motion-enhanced, multi-level deep detector and a modified Simple Online and Realtime Tracking [SORT] tracker that robustly localizes bacterial micromotors in collagen, achieving high precision with micron-scale root mean square error (RMSE), thereby supplying reliable observations required for closed-loop control in physiologically relevant matrices [78]. Pushing beyond perception, deep learning has been used to map image observations end to end to electromagnetic coil currents for automatic steering of magnetic diatom biohybrids, demonstrating vision-driven control policies that actuate real biohybrid microrobots toward targets [79,80].

##### Biomedical applications

From an application viewpoint, CI-assisted BI most directly serves in vivo diagnostics and biosensing as well as targeted drug and biologic delivery, because these application classes are frequently limited by whether biohybrid agents can be reliably localized and steered inside tissue-like, cluttered media. In tissue-mimicking collagen environments, MEMTrack enables the consistent recovery of biohybrid trajectories with micron-scale error, which directly strengthens the feasibility of closed-loop inspection and sensing tasks where the clinical value depends on maintaining a stable sensor pose and reconstructing motion states rather than merely demonstrating propulsion [78]. For delivery-style tasks at the cellular scale, deep-learning-based autonomous control of magnetic diatom biohybrid microrobots explicitly integrates real-time detection of biohybrids, obstacle cells, and target cells with path planning and tracking, thereby converting biologically enabled embodiments into repeatable, task-executable carriers for targeted interactions in biomedical microenvironments [79]. In both cases, the biomedical utility arises from the same coupling mechanism emphasized above, namely, that CI turns visually ambiguous, biologically driven agents into controllable systems by supplying perception and decision layers that remain functional under realistic imaging constraints.

Together, these studies illustrate one archetype of BI–CI synergy (CI-assisted BI), where learning enhances the observability and closed-loop controllability of biohybrid agents under degraded imaging; we now turn to the complementary archetype, BI-inspired CI, in which biological taxis motifs inform RL navigation policies.

#### BI-inspired CI

##### Definition and rationale

At the controller-design layer, recent work uses RL to rediscover chemotaxis-like behaviors for microscale robotic swimmers (Fig. 4B). Reset-free hierarchical RL yields navigation policies that mimic biased run–tumble strategies from flagella or amoebae for gradient ascent [81]. Complementarily, a sperm-chemotaxis model problem shows that RL can learn curvature modulation to climb chemical gradients, clarifying how policy structure maps to sensory noise and actuation limits [82]. These studies provide transferable priors and objectives for future BI–CI controllers, even when trained in silico.

##### Biomedical applications

From an application viewpoint, BI-inspired CI most naturally maps to targeted drug and biologic delivery, where gradient-seeking and disturbance-aware navigation are recurring primitives when continuous global imaging is limited or intermittent. Chemotactic navigation learned via reset-free hierarchical RL formalizes biological taxis as an optimization objective under partial observability and provides a computational prior that is directly aligned with repeated gradient-seeking behaviors needed for delivery-style missions in heterogeneous chemical landscapes [81]. In this way, the relevance to biomedical delivery stems from the same mechanism highlighted above, namely, that BI-derived navigation motifs are distilled into transferable computational policies that can later be composed with physical actuation and sensing constraints of microrobotic platforms.

Taken together, most systems integrate at most one CI layer with BI (perception or control) and are validated in vitro or in simulation. A fully closed-loop stack that unifies learning-based perception and policy with living actuators under clinically realistic constraints remains rare. Bridging this gap will likely require co-design of sensing, actuation fields, and learning objectives that account for biological variability while guaranteeing safety and interpretability.

### CI–HI synergy

**Fig. 5.**
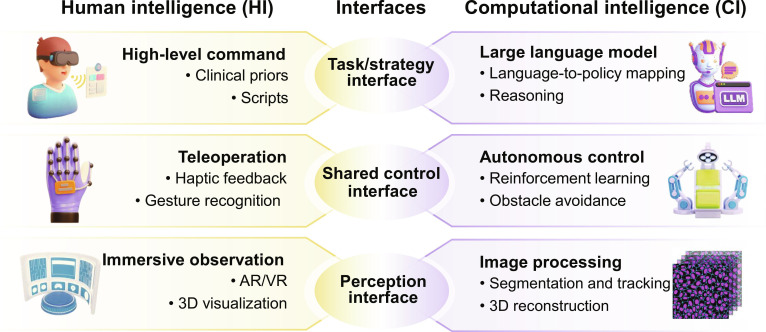
Computational intelligence (CI)–human intelligence (HI) fusion mechanisms by interface position. Task/strategy: HI provides high-level commands, clinical priors, and procedural scripts, which are fused through the task/strategy interface with CI large language models (for language-to-policy mapping and reasoning) to convert user intent into executable tasks. Shared control: HI teleoperation (haptic feedback and gesture recognition) fuses via the shared-control interface with CI autonomous control (reinforcement learning [RL] and obstacle avoidance) for coordinated microrobot actuation. Perception: HI immersive observation, augmented reality (AR)/virtual reality (VR), 3-dimensional (3D) visualization, fuses via the perception interface with CI image processing (segmentation and tracking and 3D reconstruction) to provide an immersive environment for users’ situational awareness.

When fully autonomous operation is risky or pure manual teleoperation is inefficient, the fusion of CI and HI enables intuitive and adaptive control. CI contributes perception, prediction, and algorithmic precision, whereas HI contributes situational reasoning, ethical oversight, and experience-based decision-making. This synergy is critical for image-guided interventions, targeted delivery, and remote procedures, where human supervision remains essential for safety and clinical reliability. Here, we categorize CI–HI fusion by the interface position at which HI enters the loop (Fig. 5).

#### Task/strategy interface

##### Definition and rationale

Experts express goals and priors as high-level commands (clinical scripts and constraints), and CI compiles them into executable policies. As a concrete language-to-policy example, Xu and Zhu [83] use GPT-4 with few-shot prompts to train 2 canonical microswimmers; the large language model (LLM) interprets natural-language instructions into effective propulsion strokes and achieves faster policy acquisition than a baseline RL method. In addition, LLMs have been used to generate scalar reward signals that shape training and then transfer the learned policy to hardware: a solenoid platform steers a ~500-μm ferromagnetic particle at the air–water interface under LLM-designed rewards, demonstrating promptable task shaping for physical magnetic microrobots [84]. These results indicate that language-expressed intent can be compiled by CI into field commands or policy primitives at the microscale.

##### Biomedical applications

From an application viewpoint, the task/strategy interface primarily benefits minimally invasive surgery and endovascular intervention and complex navigation-style delivery workflows within targeted drug and biologic delivery, because these scenarios often require clinicians to specify intent, safety envelopes, and procedure-level constraints while leaving low-level actuation synthesis to algorithms. Promptable, intent-expressive supervision provides a practical mechanism for compressing expert knowledge into machine-executable control primitives, which can reduce the iteration cost of designing and validating navigation behaviors before they are embedded into safety-critical, clinician-led workflows [83]. The application-level value is therefore rooted in the same synergy mechanism described above, namely, that HI supplies structured intent and priors, while CI compiles this intent into policies that can be executed under microscale physics constraints.

#### Shared control interface

##### Definition and rationale

HI provides real-time intent (via haptic devices, gestures, gaze, or joysticks), while CI arbitrates safety envelopes (collision/proximity and current/field limits) and assists with guidance. Ferro et al. [14] conducted a multiparticipant evaluation of haptic shared control for electromagnetic untethered microrobots: Combining human commands with autonomous obstacle avoidance improved safety and endpoint accuracy during 3D navigation. In networked teleoperation, model- and passivity-based haptic feedback can stabilize the channel and render distal interactions in real time, showing that reliable haptics and motion models reduce operator burden and enable precise remote micromanipulation [48]. Beyond magnetic actuation, an interactive optical-tweezer platform fuses RL planning with haptic/manual inputs and reports faster completion and better path quality than human-only or RL-only baselines, underscoring the generality of shared control for micromanipulation [47]. Finally, mixed-reality manipulation systems commonly accept multimodal operator corrections, while deep RL provides semiautonomous (shared-control) assistance to supervise navigation, exemplifying blended human intent and learned autonomy under an immersive interface [44].

##### Biomedical applications

From an application viewpoint, shared-control CI–HI interfaces are most naturally aligned with minimally invasive surgery and endovascular intervention, because these procedures remain accountability sensitive and benefit from explicit operator authority while still requiring algorithmic suppression of microscale disturbances and constraint violations. Experimental evidence on haptic shared control indicates that CI-mediated arbitration can systematically reduce unsafe motions and improve target-reaching performance, which is directly relevant to endovascular-like steering where operator intent must be preserved but assisted by real-time constraint enforcement [14]. In addition, shared-control paradigms that blend manual input with algorithmic planning in micromanipulation tasks provide a pathway for translating laboratory microrobot handling into operator-compatible workflows, which is a practical precondition for future bedside adoption even when the underlying actuation modality differs [47].

#### Perception interface

##### Definition and rationale

CI upgrades raw imaging into state estimation and situational awareness for both control and visualization, thereby reducing cognitive load. Li et al. [85] employed improved YOLOv5 to detect and track magnetic microrobots in challenging microscopy, including 2-dimensional (2D)/3D vascular models, enabling reliable constraint enforcement and closed-loop navigation. Virtual-reality (VR)-based visualization provides egocentric 3D awareness and shortens manipulation time relative to 2D displays; over the traversed regions of vascular phantoms, the mean maneuvering time was reduced by 16.4% (11.6 to 9.7 s) compared with traditional control [49]. Under clinical x-ray fluoroscopy, Alabay et al. [86] synchronized a digital twin with image-derived robot poses (~20- to 25-ms delay), yielding immersive VR that grounds human steering in anatomy, fusing CI state estimation/registration with HI intent for safer localization and guidance.

##### Biomedical applications

In terms of application domains, the perception interface is most visible in minimally invasive surgery and endovascular intervention, where intraoperative imaging constraints make it difficult for operators to maintain reliable situational awareness using raw 2D streams alone [49,86]. Under x-ray fluoroscopy, a digital-twin VR enhancement framework synchronizes image-derived robot poses into an immersive environment with an average latency of approximately 20 to 25 ms, enabling the operator to steer a magnetic robot in confined phantoms while grounding decisions in a more interpretable 3D scene [86]. This establishes a clinically relevant CI–HI loop in which CI performs registration, localization, and visual augmentation and HI retains decision authority and risk management.

In summary, CI–HI collaboration yields user-steerable microrobotic systems that combine human judgment with computational adaptability. By fusing intuitive interaction and algorithmic assistance at task/strategy, shared-control, and perception interfaces, these systems improve precision, safety, and efficiency in clinically relevant environments while preserving expert oversight.

### Higher-order synergies: Multidomain synergies in biomedical microrobots

Higher-order embodied cross-domain intelligence arises when 3 or more intelligence domains are co-designed as a coupled stack, such that embodiment, computation, and supervision jointly determine what biomedical tasks are feasible under physiological constraints. Recent progress is still uneven across synergy patterns, but several systems now demonstrate clear 3-domain coupling with in vitro or in vivo biomedical endpoints.

#### PI–BI–CI synergy

##### Definition and rationale

In microrobot swarms, PI–BI–CI synergy emerges when living effectors deliver intrinsically biological functions, yet the biomedical task demands controllable, verifiable, and repeatable outcomes. In this setting, PI establishes the physical coupling that enables batch deployment, reconfigurable gathering, and on-demand actuation in confined anatomy; BI supplies viable cell effectors that persist and function after delivery; and CI operationalizes reliability by combining imaging-aware closed-loop guidance and field/trajectory computation so that biological variability does not undermine targeting fidelity and repeatability.

##### Biomedical applications

This coupling is particularly relevant to (a) minimally invasive delivery to deep, narrow lumens and (b) targeted regenerative cell therapy where the endpoint is verified placement and retention of living therapeutics. First, endoscopy-enabled rapid deployment combined with imaging-aware magnetic navigation allows stem-cell spheroid microrobots to be delivered as a controllable cohort to otherwise hard-to-access luminal sites: CI (real-time endoscopic/ultrasound verification and guidance) structures PI (magnetic actuation) into a reliable, stepwise delivery workflow, while BI preserves the viability and therapeutic potential of the delivered cell spheroids at the target region [87]. Second, in joint repair, a cohort of mesenchymal-stem-cell-loaded microrobots can be guided in 3 dimensions by an electromagnetic actuation system and then stably retained by magnetic fixation; here CI (field/trajectory computation and compatible visualization during targeting) amplifies PI (3D magnetic steering and fixation) to overcome injection-induced dispersion, enabling reproducible on-site delivery of viable mesenchymal stem cell effectors (BI) for cartilage regeneration [88].

#### PI–CI–HI synergy

##### Definition and rationale

PI–CI–HI synergy is typically realized through an image-guided feedback loop in which the microrobot embodiment is engineered for stable field transduction and visibility, computational components support localization and procedure-level planning/navigation under sensing constraints, and the human operator specifies goals and enforces safety boundaries within clinically compatible workflows. In this synergy, the physical design sets the operating limits and imaging quality, so it ultimately determines what localization, guidance, and shared control can achieve reliably.

##### Biomedical applications

A representative example is an imaging-guided bioresorbable acoustic hydrogel microrobot platform, which couples a biodegradable hydrogel body (PI) with acoustic actuation and real-time imaging feedback (CI) to enable targeted operation in vivo under operator supervision (HI) [76]. In a bladder tumor setting, the platform demonstrates task-level therapeutic relevance by enabling localized microrobotic access and treatment associated with tumor suppression while maintaining an explicit biodegradation pathway that directly addresses posttreatment residue concerns. Complementarily, clinical MRI-guided navigation with computational posture/steering planning improves target-lobe distribution and target-reaching outcomes under interventional workflows, translating imaging and planning into clinically interpretable targeting efficiency [77]. This class of PI–CI–HI systems maps most naturally to minimally invasive surgery and endovascular intervention and targeted intraluminal therapy, where the requirement is not maximal autonomy but dependable execution with accountable supervision under real imaging and safety constraints [76,77].

### Comparative summary

Table 2 provides a compact, task-oriented scorecard of representative cross-domain synergies in biomedical microrobotics. For each study, we summarize the targeted biomedical task and the synergy-driven advance and report evidence-based ratings across 6 assessment dimensions: sensing/observability (S), control fidelity (C), autonomy/adaptation (A), clinical workflow compatibility (W), biosafety/clearance (B), and system integration and task closure (I).

**Table 2.** Summary of representative cross-domain synergies in biomedical microrobots, highlighting synergy-enabled advances and evidence-based ratings across 6 assessment dimensions

Synergy	Targeted issue	Advancement	Assessment dimensions						Ref.
			S	C	A	W	B	I	
PI–BI	Targeted chemo–photothermal tumor therapy	Magnetic biohybrid enables on-demand drug release	⋆	⋆ ⋆	—	⋆	⋆ ⋆	⋆ ⋆	[67]
	Remote immunotherapy of tumor spheroids	Immune-cell effectors + magnetic steering yield a strong antitumor endpoint	⋆ ⋆	⋆ ⋆	⋆	⋆ ⋆	⋆ ⋆ ⋆	⋆ ⋆	[30]
PI–CI	Autonomous 3D magnetic microrobot positioning	Reinforcement learning improves micron-scale positioning under nonlinear actuation	⋆ ⋆	⋆ ⋆ ⋆	⋆ ⋆ ⋆	⋆	—	⋆	[69]
	Adaptive ultrasound navigation in microfluidic channels	Model-based reinforcement learning enables sample-efficient adaptation to new channels	⋆ ⋆	⋆ ⋆	⋆ ⋆ ⋆	⋆	—	⋆	[46]
	Closed-loop ultrasound microswarm path following	Video-rate tracking + reinforcement learning supports closed-loop trajectory execution	⋆ ⋆ ⋆	⋆ ⋆ ⋆	⋆ ⋆ ⋆	⋆	—	⋆	[70]
	Imaging-guided in vivo bladder tumor therapy	Bioresorbable drug depot + imaging guidance translates navigation into a therapy endpoint	⋆ ⋆ ⋆	⋆ ⋆	⋆ ⋆	⋆ ⋆ ⋆	⋆ ⋆ ⋆	⋆ ⋆ ⋆	[76]
BI–CI	Tracking biohybrid microrobots in dense collagen	Learning-based tracking improves observability in low-contrast biological scenes	⋆ ⋆ ⋆		⋆	⋆ ⋆	—	⋆	[78]
CI–HI	Haptic shared control of multiple microrobots	Shared control reduces operator burden while improving speed and accuracy	⋆ ⋆	⋆ ⋆	⋆ ⋆	⋆ ⋆ ⋆	—	⋆	[14]
	VR-enhanced x-ray localization and steering	Low-latency virtual enhancement improves situational awareness under fluoroscopy	⋆ ⋆ ⋆	⋆ ⋆	⋆ ⋆	⋆ ⋆ ⋆	—	⋆	[86]
PI–BI–CI	Endoscopy-assisted endoluminal delivery of living spheroids	Endoscopy verification + magnetic steering enable rapid, verifiable living delivery	⋆ ⋆ ⋆	⋆ ⋆ ⋆	⋆	⋆ ⋆ ⋆	⋆ ⋆	⋆ ⋆ ⋆	[87]
	In vivo knee-joint targeting for cartilage regeneration	CI-informed actuation boosts on-target placement of living therapeutics in vivo	⋆	⋆ ⋆	⋆ ⋆	⋆ ⋆ ⋆	⋆ ⋆ ⋆	⋆ ⋆ ⋆	[88]
PI–CI–HI	MRI-guided microrobot navigation in hepatic arteries	Planning + clinical MRI steering increases targeting efficiency at human scale	⋆ ⋆ ⋆	⋆ ⋆	⋆ ⋆	⋆ ⋆ ⋆	⋆	⋆ ⋆	[77]

PI, physical intelligence; BI, biological intelligence; CI, computational intelligence; HI, human intelligence; 3D, 3-dimensional; VR, virtual reality. Dimensions: S, sensing/observability; C, control fidelity; A, autonomy/adaptation; W, clinical workflow compatibility; B, biosafety/clearance; I, system integration and task closure. Ratings reflect the strength of task-relevant evidence rather than cross-paper numerical comparability: ⋆⋆⋆, strong task-relevant evidence; ⋆⋆, clear but partial evidence; ⋆, proof-of-concept or weak evidence; —, not reported (stars are shown vertically).

**Fig. 6.**
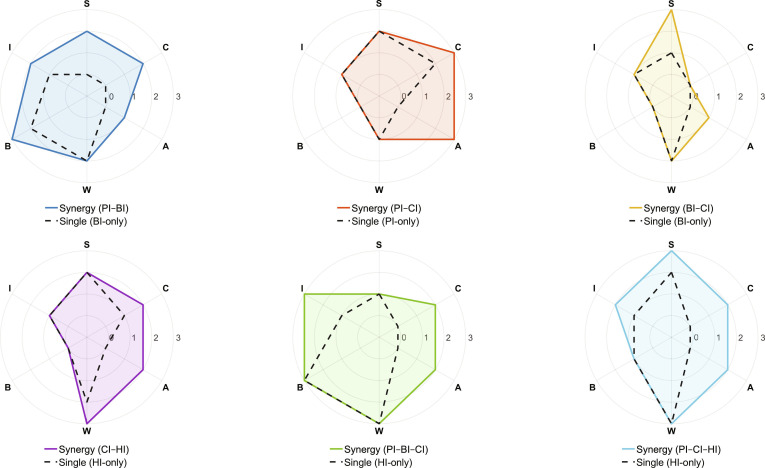
Cross-domain synergy versus single-domain baselines visualized as evidence-based radar profiles across 6 assessment dimensions (S, sensing/observability; C, control fidelity; A, autonomy/adaptation; W, clinical workflow compatibility; B, biosafety/clearance; I, system integration and task closure). Scores range from 0 to 3, where 0 denotes the inner ring (not reported/insufficient task-relevant evidence) rather than the origin. Solid polygons indicate representative cross-domain synergy systems (PI–BI, PI–CI, BI–CI, CI–HI, PI–BI–CI, and PI–CI–HI), while dashed polygons indicate the closest single-domain baselines under matched task settings. PI, physical intelligence; BI, biological intelligence; CI, computational intelligence; HI, human intelligence.

To make the benefits of cross-domain intelligence more intuitive under the field’s nonunified benchmarking landscape, Fig. 6 further compares each synergy profile with a matched single-domain baseline, prioritizing within-study ablations or explicitly defined control conditions, so that cross-domain gains are interpreted under comparable task settings using the same 6 dimensions as in Table 2.

In PI–BI systems, the advantage is expressed through the simultaneous satisfaction of transport efficiency and payload constraints that typically compete at the microscale. In AlgaeSperm, rapid locomotion is paired with a high drug loading and a time-bounded release readout, so the reported 2.3 body lengths/s, 91.4% doxorubicin loading, and 54.2% release within 3 h jointly indicate that the carrier can both reach the target region and deliver a meaningful dose on a therapeutically relevant time scale [67]. In immune-cell microrobots, the biological component is reflected most transparently at the outcome level. The 0.8% viability of 3D tumor spheroids after 24 h provides a direct efficacy endpoint, showing that cell-based functionality, together with guided positioning and monitoring, translates into strong antitumor action in a 3D tissue-like model rather than only in simplified 2D assays [30]. Importantly, the same study reports corresponding immune-cell-only controls (without magnetic actuation/steering), allowing the PI–BI synergy to be interpreted as an end-to-end upgrade from “cell function alone” toward controllable delivery that translates into stronger task closure (Fig. 6, notably in C/W/I).

In PI–CI systems, the reported gains concentrate on controllability and robustness under nonlinear and uncertain actuation, where purely model-based control can be brittle and purely passive design cannot adapt. For magnetic 3D positioning, a mean distance error below 30 μm after 4 control steps indicates that learned policies can rapidly converge to precise positioning within a millimeter-scale workspace, which is critical for microscale interventions near anatomical boundaries [69]. Crucially, Abbasi et al. explicitly compared RL against a classical controller (proportional–integral–derivative), enabling a matched “PI-only/conventional control” baseline and making the PI–CI advantage interpretable as increased autonomy/adaptation and sustained control fidelity under the same hardware and sensing pipeline (Fig. 6, strongest contrast in A and C). For ultrasound-driven navigation across microfluidic channels, the combination of 90% success within 1 h and the improvement from 50% to over 90% with 30 min of additional training makes the advantage interpretable as fast adaptation to new environments, a practical requirement when geometric and flow conditions vary across patients or phantoms [46]. Closed-loop feasibility is further grounded by the perception bandwidth needed for feedback control. Tracking swarms at approximately 33 fps down to 10 μm implies that sensing and control can operate at video rate on small collectives, supporting real-time corrections rather than open-loop execution [70]. When these control capabilities are coupled to in vivo therapy, the approximately 93% reduction of bioluminescence by day 14 provides a task-level readout that the actuation and guidance pipeline is not only mechanically functional but also therapeutically effective in a realistic biological context [76].

In BI–CI synergy, the primary bottleneck is that biological environments are visually degraded, dynamic, and cluttered, so perception quality becomes the limiting factor for downstream autonomy. Precision and recall quantify false positives and missed detections, while RMSE quantifies localization fidelity. The reported 76% precision, 51% recall, and 1.84 μm RMSE in collagen collectively indicate that state estimation remains usable even in dense, low-contrast scenes, which is a prerequisite for reliable closed-loop control, safety constraints, and reproducible evaluation [78]. Notably, the same work benchmarks against classical tracking baselines (e.g., TrackMate), thus providing a matched “BI-only perception pipeline” baseline and demonstrating that BI–CI synergy primarily expands observability (Fig. 6).

For CI–HI synergy, the advantage is reflected in operational efficiency and stability of interaction when full autonomy is not yet reliable. Reduced travel time together with improved positioning accuracy indicates that shared control can offload continuous manual corrections while preserving operator intent [14]. Because the paper reports a manual-only condition under the same task setup, the CI–HI benefit can be read directly as a synergy-over-HI baseline (Fig. 6, gains primarily in C/A/W). Likewise, low system latency at video rate constrains feedback delay to a range compatible with responsive HITL manipulation in fluoroscopic settings [86].

Higher-order synergies illustrate how combining 3 domains can shift improvements from isolated component performance to end-to-end task execution and selectivity. For PI–BI–CI in deep endoluminal swarm delivery, integrating physically actuated steering with protocol-level coordination and imaging verification enables a living spheroid swarm to traverse extended anatomical routes within minutes, quantified by a ~100-cm targeted delivery that is completed in <8 min [87]. For PI–BI–CI in knee-joint swarm targeting, CI-informed electromagnetic actuation markedly improves task-level placement compared with injection alone, raising targeting to ≥80% versus an injection-only baseline that can drop to 1.5% [88]. This within-study comparison makes the integrative advantage scenario faithful: the gain is not a generic key performance indicator boost but a task-closure jump driven by adding controllable physical steering and decision logic to an otherwise single-domain delivery (Fig. 6, strongest in W/I and C). For PI–CI–HI, human-scale mixed-reality navigation in hepatic arteries reports explicit controls without magnetic field (i.e., standard injection workflow), enabling a matched HI-only baseline within the same study [77]. Under this matched comparison, posture planning and MRI steering translate into clinically interpretable targeting shifts and increased target hits, consistent with synergy expanding both workflow compatibility and task closure beyond what manual injection alone can guarantee (Fig. 6). Taken together, the table clarifies how different synergies map to different, task-relevant indicators, and it naturally motivates Challenges by highlighting the need for benchmarking frameworks that move toward comparable task-level endpoints while remaining faithful to scenario-dependent constraints.

## Challenges

**Fig. 7.**
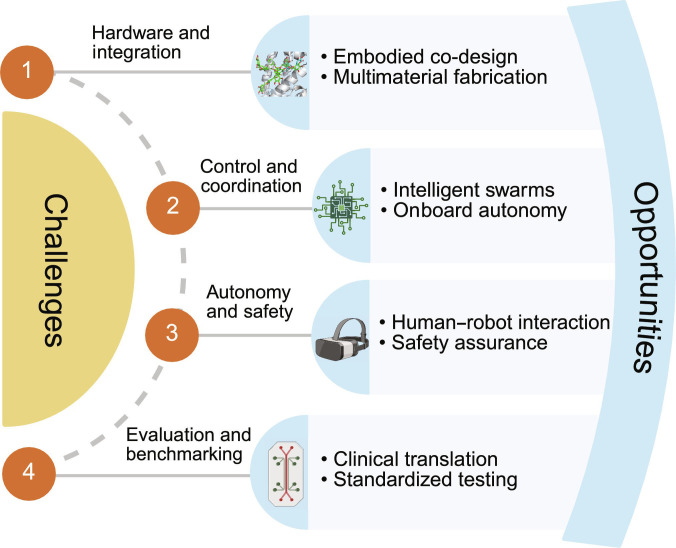
Integrated visual framework summarizing the key challenges and future research directions in advancing microrobot integration for medicine, with each challenge theme explicitly aligned to the intelligence domains and cross-domain interfaces that most strongly constrain progress.

Integrating diverse forms of intelligence into microrobots presents major challenges. Within the PI/BI/CI/HI framework, these 4 challenge themes primarily reflect bottlenecks at specific interfaces: hardware and integration (PI–BI and PI–CI), control and coordination (PI–CI and BI–CI), autonomy and safety (CI–HI under PI/BI constraints), and evaluation and benchmarking (cross-domain). This section outlines key obstacles in engineering, computation, and clinical translation, along with future research directions. Fig. 7 maps 4 core challenge areas to their related research opportunities and guides the structure of the following subsections.

1. Hardware and integration challenges: Multifunctional integration remains a major bottleneck for biomedical microrobots [3,50]. In cross-domain terms, this bottleneck is most acute at the PI–BI interface and the PI–CI interface, where responsive materials, magnetic/electronic elements, and living components must be physically integrated while preserving both physiological compatibility and control authority. Most current designs offer a single function, smart actuation or biohybrid behavior, as integrating both in a 1-μm-scale system is highly complex. Components are often incompatible: a stimulus suitable for polymers may harm cells, and miniaturized electronic or magnetic modules can introduce toxicity [89,90]. These material and biocompatibility constraints are further compounded by fabrication and scale-up limitations. Multidomain robots require hybrid workflows, including top-down lithography, nanoscale self-assembly, and cell culture, which are hard to unify into a single reproducible pipeline and remain difficult to scale up beyond small-batch prototyping [3,91]. Most prototypes are manually assembled, which limits reproducibility [6,92]. Without integrated autonomy, these systems are sensitive to environmental changes [93]. Overcoming these barriers is essential: in vivo intelligent behavior demands robust integration of materials, biology, and electronics, along with scalable manufacturing to transition from fragile prototypes to clinically viable systems [50].
2. Control and coordination: Coordinating microrobots with multiple intelligences remains a core challenge [3,94]. This challenge primarily reflects PI–CI and BI–CI coupling: PI determines field-to-motion transduction and time-varying body dynamics, BI introduces heterogeneity and stochasticity, and CI must deliver robust perception and control that remain valid beyond idealized in vitro conditions. These systems exhibit complex dynamics from PI, CI, and BI components. Classical control struggles with the nonlinear, time-varying behavior of smart materials and biohybrids [78]. While RL offers promise, its reliance on large datasets and trial and error makes it impractical in vivo [46,70]. Simulations can help, but accurately modeling coupled fluid, chemical, and biological processes remains computationally intensive and limited [95,96]. Robust performance under dynamic conditions is still lacking [3,50]. Swarm control poses added challenges: visual and chemical signaling introduce noise and uncertainty [97–99]. Bacteria-inspired chemical communication (BI) must be integrated with CI algorithms to interpret and act on messages, which is an unresolved task. Transferring controllers from in vitro benchmarks to in vivo environments typically requires substantial adaptation, as imaging noise, flow disturbances, and tissue interactions impose constraints that are absent in simplified test beds [93]. Advancing control and communication strategies is critical for reliable microrobot autonomy in complex environments.
3. Autonomy and safety in medical environments: Advancing autonomy in microrobots requires high confidence in decision-making, with safety and ethical accountability being critical for clinical use. Within the PI/BI/CI/HI framework, autonomy is constrained not only by CI decision-making but also by PI/BI biosafety requirements and therefore depends strongly on CI–HI coupling to preserve accountability through shared supervision and clinically interpretable state awareness. Regulatory bodies will demand deterministic behavior or fail-safe systems [3,37,50]. As Yang et al. [100] note, high-autonomy robots essentially perform medical acts, raising significant regulatory issues. AI errors in vivo can cause immediate harm, making HI supervision essential in the near term [3]. However, managing large microrobot swarms is still challenging; without intelligent interfaces like AR or AI-assisted controls, clinicians may face cognitive overload [37,93]. Biocompatibility and posttreatment retrieval also pose safety concerns. Nonbiodegradable robots, especially those with metals or electronics, risk chronic toxicity or immune reactions [89]. PI approaches, such as time-triggered dissolution, offer partial solutions, but ensuring complete clearance or safe degradation remains a research priority [76]. These challenges define the current boundaries of safe autonomy in medical applications.
4. Evaluation and benchmarking: There is still no clear method to rigorously quantify intelligence or performance in micro/nanorobots [3,37]. Because performance is a coupled outcome of PI, BI, CI, and HI, benchmarking must report task-level metrics together with domain-resolved failure modes, so that limitations at a specific interface do not masquerade as system-level unreliability. To make evaluation more actionable, the field can adapt established frameworks from macroscale medical robotics. For example, adopting a “levels-of-autonomy” framework allows researchers to classify capabilities from teleoperation (low-level) to full autonomy (high-level) [100]. Robotics typically relies on task benchmarks such as navigation error or retrieval success rate; however, when biological components are involved, outcomes may be stochastic [6]. Therefore, benchmark tasks should evolve from simple traversing micromazes to standardized vascular phantoms and organ-on-a-chip platforms that replicate physiological complexity [30,101]. Metrics should also shift from physical parameters to task-level indicators, such as delivery efficiency and off-target deposition rates. Multidomain systems also exhibit coupled biological, material, and algorithmic failure modes, which complicates causal attribution [6]; without common metrics and systematic failure-mode analysis, it is hard to build clinical trust, underscoring the need for dedicated safety, regulatory, and evaluation frameworks [50].

Despite current challenges, they reveal opportunities for progress. Advances in integration, control, and safety can evolve microrobots into reliable medical tools. Improvements in manufacturing, algorithms, and regulation will support clinical translation. The following sections outline how cross-domain intelligence may overcome key barriers.

### Future direction I: Embodied design and manufacturing

Overcoming integration barriers will require embodied AI frameworks where morphology, materials, and control coevolve via computational optimization [94,102,103]. Crucially, this paradigm must prioritize material life cycle management to ensure biocompatibility. Future multimaterial fabrication is expected to enable the encapsulation of functional cores within bio-inert or immunomodulatory shells [66], while “degradation by design” must be integrated as an optimization constraint to guarantee safe clearance (e.g., enzymatic dissolution) [76]. On the manufacturing front, converging high-resolution additive techniques (e.g., digital light processing and multimaterial 2-photon lithography) [104–107] with molecular assembly (e.g., DNA origami) [108] will enable the direct construction of these safe, multifunctional systems, accelerating the transition from trial-and-error prototyping to predictable clinical engineering [102].

### Future direction II: Intelligent swarms and onboard autonomy

To address control and coordination challenges, distributed and hierarchical architectures are emerging as promising solutions [3,97]. Individual robots are equipped with local sensing and lightweight decision-making for real-time responsiveness [73], while swarm-level coordination relies on rule-based strategies for task allocation and coherent behavior [97]. Synthetic biology offers a complementary path: engineered gene circuits or DNA computing enable cells to autonomously respond to biochemical cues [109]. Increasing integration of microelectronics and biocomputation is expected to enhance autonomy and robustness [110], allowing swarms to handle routine tasks, such as obstacle avoidance and formation control, without human input [97,111].

### Future direction III: Human–robot shared control and safety assurance

Ensuring medical autonomy and safety requires a shared-control model. AR/VR overlays and haptic feedback enhance the perception of microscale interactions, while intent guidance and constraint-based assistance ease operators’ workload [44,47,48]. For swarm teleoperation, efficient data aggregation and control dispatch mitigate cognitive overload [112]. System-level safety includes transparent monitoring, anomaly alerts, and emergency abort protocols [50]. Hardware development should focus on recyclable, biodegradable designs to ensure zero residue postoperation, enabling safer autonomy in clinical use [3,76].

### Future direction IV: Clinical translation and validation

Future progress will depend on translating multi-intelligent microrobots into clinically relevant systems, from proof of concept to first-in-human use as the technology readiness level matures, a process that extends beyond technical validation to encompass regulatory and ethical frameworks [3,50]. As microrobots evolve into “combination products” integrating living cells, smart materials, and autonomous algorithms, the regulatory pathway and risk class vary across jurisdictions (e.g., US Food and Drug Administration device classes I to III and European Union Medical Device Regulation IIb/III), challenging existing regulatory categories and necessitating new standards for safety and ethical accountability, particularly regarding clear responsibility and human oversight for autonomous decisions [3,100]. To support this translation, current efforts focus on efficacy studies in animal models and standardized benchmarks, such as vascularized lab-on-a-chip platforms [76,113], while progressing toward standard-aligned verification (e.g., biocompatibility, sterility, and clearance) to build submission-grade evidence. Crucially, digital-twin frameworks combining high-fidelity simulation with experimental data will support regulatory review by stress-testing microrobots under worst-case scenarios, turning model–experiment discrepancies into traceable signals for reliability improvement and auditable safety cases [3,37].

## Conclusion

This review introduces embodied cross-domain intelligence as a framework for biomedical microrobots, explaining how PI, BI, CI, and HI jointly support adaptive and safe behavior in complex biomedical tasks. We surveyed mechanisms and interfaces that implement these domains, from biohybrid embodiments and physics-embedded learning to shared-control and perception interfaces that couple automation with clinical expertise, and we further mapped these synergies to representative biomedical application classes. Recent systems are beginning to combine multiple domains within a single workflow, suggesting a realistic path toward clinically accountable cross-domain synergy. Across these studies, we identified long-standing barriers in integration and fabrication, robust control under physiological constraints, biocompatibility and safety, and quantitative evaluation and benchmarking. Progress in embodied co-design, distributed control, swarms, digital twins, and standardized validation should enable microrobots that move from single-intelligence prototypes toward dependable, multi-intelligent systems integrated into future diagnostic and therapeutic workflows.
